# Spontaneous Cerebrospinal Fluid Rhinorrhea in an Otherwise Healthy 42-Year-Old Female Presenting to a Resident Clinic

**DOI:** 10.7759/cureus.104924

**Published:** 2026-03-09

**Authors:** Andrew W Antes, Nancy V Nguyen, Kelty C White

**Affiliations:** 1 Family Medicine, Dominican Hospital, Capitola, USA; 2 Family Medicine Residency Program, Methodist Hospital of Sacramento, Sacramento, USA; 3 Family Medicine, Sierra Nevada Family Medicine Residency Program, Grass Valley, USA

**Keywords:** atraumatic cerebrospinal fluid rhinorrhea, differential diagnosis of rhinorrhea, differential diagnostic process, endoscopic csf leak repair, resident, resident clinic, spontaneous cerebrospinal fluid leak, spontaneous cerebrospinal fluid rhinorrhea, traumatic cerebrospinal fluid rhinorrhea

## Abstract

Rhinorrhea, a common symptom documented in patients presenting to primary care clinics, urgent care facilities, and emergency departments, is often attributed to viral, bacterial, or allergic causes when no other obvious etiology, such as a foreign body, is found. However, in the absence of other infectious signs or symptoms, cerebrospinal fluid (CSF) rhinorrhea should be considered more prominently in a physician’s differential diagnosis than it often is. This process can be either traumatic or non-traumatic, so even in the absence of recent head trauma, it should be evaluated, as this also leaves the patient vulnerable to multiple life-threatening complications. In this report, we discuss one such patient who presented to multiple physicians with progressively worsening rhinorrhea, which was discovered to be due to a CSF leak through a defect in her cribriform plate and the treatment she received to resolve this issue once it was identified.

## Introduction

Cerebrospinal fluid (CSF) rhinorrhea is a condition in which the fluid surrounding a patient’s brain, known as CSF, leaks out of the intracranial cavity into the nasal passages, causing clear nasal discharge that exits the nares and resembles infectious or allergic rhinorrhea. While it may present similarly, unlike infectious rhinorrhea, CSF rhinorrhea requires urgent hospitalization and management because the potential for life-threatening complications, such as meningitis from ascending infections, is significant if left untreated [1,2]. Traumatic CSF rhinorrhea can often be localized to specific intracranial injuries, whereas atraumatic cases may require further investigation to identify the site of the leak. A study of patients with CSF rhinorrhea in Malaysia, Singapore, and Thailand found the cribriform plate to be the most common site of CSF leakage [1]. Compared with traumatic CSF rhinorrhea, spontaneous or non-traumatic cases were estimated to account for only 28% of cases in a recent systematic review [3], although other studies published in recent years suggest that atraumatic CSF rhinorrhea may actually be more common than traumatic cases [1,4].

## Case presentation

The patient was a 42-year-old female with no significant past medical history. She had initially presented to an urgent care clinic with a two- to three-day history of cough and tested negative for coronavirus disease 2019 (COVID-19) and influenza. One week after symptom onset, she presented to the emergency room for evaluation of clear, unilateral right-sided rhinorrhea that had persisted for one week, along with a dry cough, without any additional symptoms. The physical exam was unremarkable, and no laboratory tests or imaging studies were obtained. She was diagnosed with allergic rhinitis and considered stable for discharge. She was given recommendations for over-the-counter allergy treatments, including fluticasone nasal spray, saline sinus rinses, warm fluids such as tea with honey, adequate oral hydration, use of a bedroom humidifier, warm showers or baths, and follow-up with her primary care provider.

Seven days after her emergency department visit, she presented to her primary care provider, a resident physician at a family medicine residency clinic. At the time of this visit, she required a face mask to secure a washcloth to her face to staunch the continuous flow of clear fluid from her right nostril. She had developed daily headaches with no clear association with movement, time of day, or any other palliative or provoking factors. She continued to deny any allergy symptoms, such as itchy eyes or throat, sneezing, or wheezing. She had no history of recent head injury, falls, or trauma, and stated that the nasal fluid tasted salty.

She had been taking cetirizine and pseudoephedrine without improvement. The physical exam was notable for copious unilateral right-sided rhinorrhea, causing a puddle of fluid at her feet during the visit. There were no meningeal signs, sinus tenderness, conjunctival injection, or other signs of allergic reaction observed on exam. Both the resident physician and the attending physician had significant concerns for a CSF leak due to the persistent unilateral rhinorrhea and advised the patient to go immediately to the local emergency room for evaluation. The emergency room on-call physician was contacted, and suspicions were communicated to guide workup and treatment.

**Figure 1:**
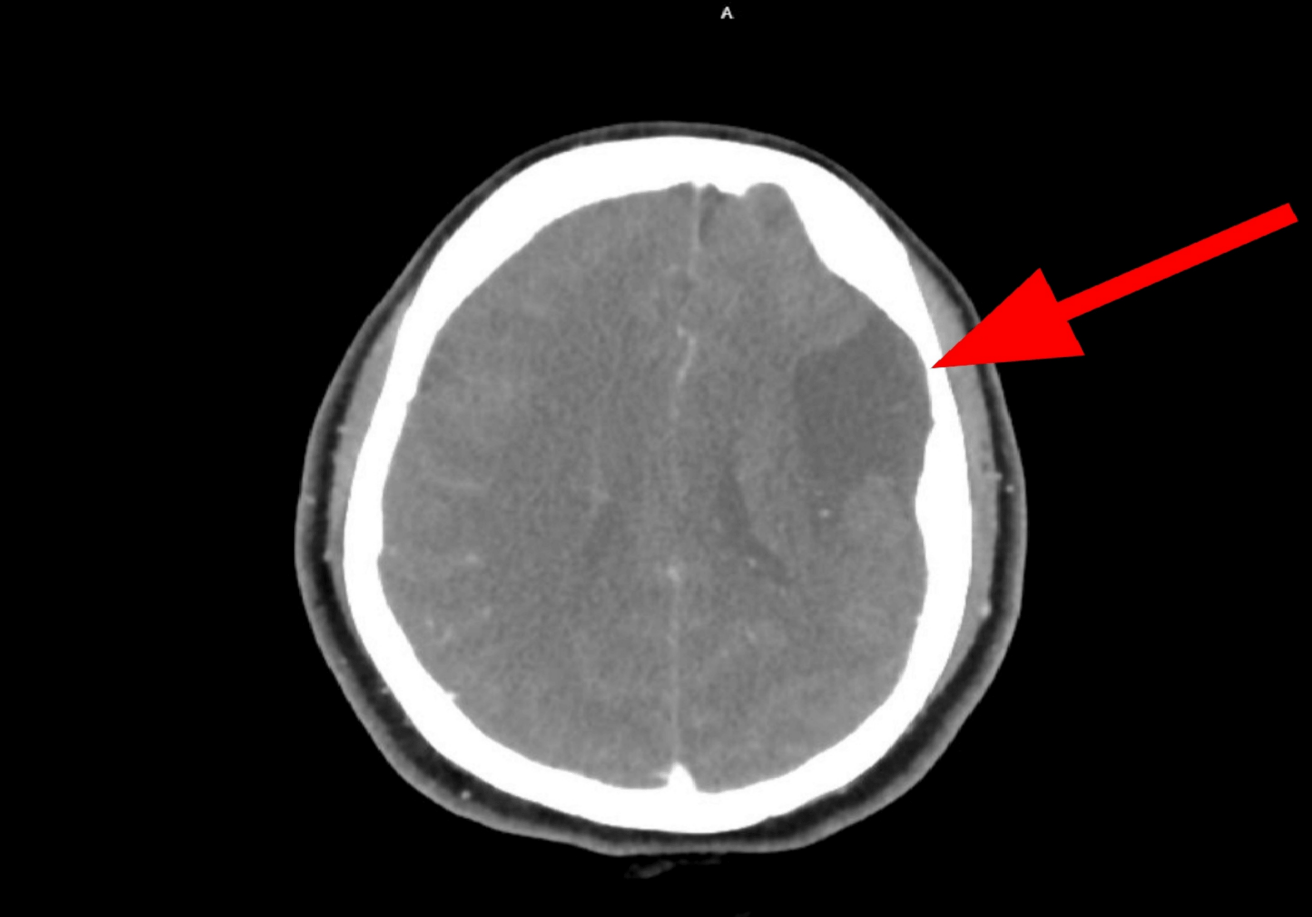
CT image showing a possible previous infarct in the left frontal lobe (red arrow) with no other abnormalities detected CT: computed tomography

In the emergency department, a sample of nasal fluid was collected to test for beta-2 transferrin. A CT sinus with contrast showed a moderate-sized region of encephalomalacia involving the left frontal lobe, consistent with a prior infarct as seen in Figure 1, but was otherwise unremarkable, with no evidence of fracture, trauma, or other abnormality. Lab work at that time revealed leukocytosis of 16.1 K/uL (0.02 ×10⁹/L) and an elevated platelet count of 433 K/uL (433 ×10⁹/L) (Table 1). With the CT negative for acute abnormalities and the leukocytosis, which the ED physician believed might be reactive to the patient's prior upper respiratory infection (URI), the patient was discharged home. She was prescribed amoxicillin-clavulanate 875-125 mg BID for 10 days and oxymetazoline nasal spray BID for three days for possible sinusitis vs rhinosinusitis. Three days later, the beta-2 transferrin results came back positive, and both the emergency department physician and a family medicine resident contacted the patient. She was instructed to go to a nearby tertiary center to be evaluated emergently by Neurosurgery for a suspected non-traumatic CSF leak.

**Table 1:** Initial ED lab results Initial ED lab work drawn after concern for CSF leak was raised by PCP, including beta-2 transferrin, which later came back positive ED: emergency department; CSF: cerebrospinal fluid; PCP: primary care provider

Variable	Patient value	Reference range
White blood cells (K/uL)	16.1	4.3 - 11.0
Red blood cells (million/uL)	4.32	4.00 - 5.40
Hemoglobin (gm/dL)	12	12.0 - 16.0
Hematocrit (%)	36.2	36.0 - 47.0
Mean corpuscular volume (fL)	83.8	80.0 - 100.0
Mean corpuscular hemoglobin (pg)	27.8	25.0 - 35.0
Mean corpuscular hemoglobin concentration (gm/dL)	33.1	31.0 - 37.0
Red cell distribution width (%)	13.4	11.5 - 14.5
Platelets (K/uL)	433	150 - 400
Neutrophils (%)	74	-
Lymphocytes (%)	20	-
Monocytes (%)	4	-
Eosinophils (%)	1	-
Basophils (%)	1	-
Absolute neutrophils (K/uL)	11.9	1.3 - 7.4
Absolute lymphocytes (K/uL)	3.2	0.7 - 3.1
Absolute monocytes (K/uL)	0.6	0.2 - 0.9
Absolute eosinophils (K/uL)	0.2	0.0 - 0.4
Absolute basophils (K/uL)	0.1	0.0 - 0.1
Estimated creatinine clearance (mL/min)	75.91	-
Creatinine (mg/dL)	0.73	0.50 - 1.50
Estimated glomerular filtration rate (mL/min/1.73 m^2^)	105	≥90
Sodium (mmol/L)	138	136 - 146
Potassium (mmol/L)	4.1	3.5 - 5.5
Chloride (mmol/L)	103	98 - 110
CO_2_ (mmol/L)	26	24 - 32
Anion gap (mmol/L)	13	4 - 22
Glucose level (mg/dL)	105	70 - 99
Blood urea nitrogen/creatinine ratio	16.4	8.0 - 24.0
Calcium (mg/dL)	8.9	8.4 - 10.5
Alanine aminotransferase (IU/L)	10	0 - 55
Aspartate aminotransferase (IU/L)	9	5 - 34
Albumin (gm/dL)	4	3.0 - 5.0
Alkaline phosphatase (IU/L)	80	40 - 150
Total bilirubin (mg/dL)	<0.3	0.2 - 1.2
Corrected calcium (mg/dL)	8.9	8.4 - 10.5
Protein total (gm/dL)	8.1	6.0 - 8.0
Globulin (gm/dL)	4.1	-
Albumin/globulin ratio	1	-
Beta-2 transferrin	Detected	-

Upon arrival at the tertiary center the next day, the patient reported continued episodes of nausea and vomiting, most recently several hours before presentation, as well as blurry vision and bitemporal, pressure-like headaches. A physical examination performed by the tertiary center ED physician revealed rhinorrhea and midline cervical spine tenderness, but the exam was otherwise unremarkable. The patient was subsequently admitted, and the ENT, Neurosurgery, and Ophthalmology teams were consulted. Ophthalmology evaluated the patient before surgery. Intraocular pressure (IOP) was normal, and the exam was negative for papilledema.

The patient was taken to surgery on day two of admission, where the surgical team performed a right maxillary antrostomy, right total ethmoidectomy, right sphenoidotomy, right frontal sinusotomy, right endoscopic CSF leak repair, and CT-guided stereotactic navigation. The operative note reported a skull base defect, a small encephalocele, and a CSF leak grossly identified at the right cribriform plate/olfactory groove. The encephalocele was bipolared with good recession, and the defect was repaired with Cook extracellular matrix, a middle turbinate mucosal graft, and Avitene/Surgicel/NasoPore packing. The patient was discharged on acetazolamide 500 mg SR BID for 30 days. She was also referred to Neuro-ophthalmology and has been followed up by her family medicine physician at the residency clinic for regular follow-up.

## Discussion

Our patient presented to a residency clinic with a life-threatening condition after previous evaluations in urgent care and the emergency department for similar symptoms in the preceding days. While it is unclear whether her rhinorrhea was initially infectious and later progressed to CSF rhinorrhea or represented CSF rhinorrhea from the beginning, it is notable that the diagnosis was not considered until her third healthcare visit. Fortunately, the correct diagnosis was made, and the patient received appropriate treatment. If imaging or evaluation of the nasal discharge had been performed during her earlier visits, her diagnosis might have been established up to two weeks sooner, significantly reducing her risk of life-threatening complications. The differential diagnosis for rhinorrhea is broad, and infectious causes are most common. However, potentially life-threatening conditions must also be considered and ruled out. This is especially important in cases without the typical symptoms or examination findings that suggest more common causes, such as allergies, infections, or foreign bodies.

CSF rhinorrhea cases are generally classified as traumatic or non-traumatic. Non-traumatic cases are often secondary to intracranial processes such as meningitis, mass lesions, or intracranial hypertension, while traumatic cases are further classified as iatrogenic or non-iatrogenic [1]. Risk factors for spontaneous CSF rhinorrhea include female sex, increased intracranial pressure, obesity, and obstructive sleep apnea [5,6]. Although non-traumatic CSF leaks were previously estimated to account for only about 28% of cases, multiple recent studies suggest that atraumatic CSF leaks may actually be more common than traumatic CSF leaks [1,3,4]. Therefore, any patient with persistent clear rhinorrhea should be evaluated for a CSF leak, regardless of trauma history.

High-resolution CT of the skull base, paranasal sinuses, and temporal bone structures without contrast is considered the first-line imaging choice because it is non-invasive, provides satisfactory diagnostic information, and is relatively low-cost. If CT is unavailable or inadequate, a brain MRI without contrast can be considered as a second-line option, offering greater diagnostic accuracy but at a higher cost [1,3]. Imaging often provides critical information for the treatment of CSF leaks, depending on their type and location. However, in cases similar to the one described above, where imaging is inconclusive, fluid testing for the presence of CSF can provide vital diagnostic information. If CSF is confirmed but no leak origin is visible on CT or MRI, endoscopic evaluation by a surgical team may be necessary to locate and repair the leak. Endoscopic repair of CSF leaks is recommended as first-line treatment [6]. If a patient is not a surgical candidate or surgical repair is not feasible, medical management of CSF rhinorrhea is recommended. The goal of medical management is to reduce intracranial pressure and CSF flow through the defect to allow healing, using interventions such as drains, diuretics, or bed rest [4].

## Conclusions

CSF rhinorrhea can mimic more common, less serious conditions such as allergic rhinitis or viral upper respiratory infections. However, persistent, clear unilateral rhinorrhea should prompt consideration of a CSF leak because of the potentially life-threatening nature of the condition and its complications. Evaluation is warranted even in patients without trauma or known risk factors, given the frequency of idiopathic and non-traumatic CSF rhinorrhea. Earlier recognition and consideration of this diagnosis can reduce delays in care, minimize complications, and save lives.
